# Association between NDUFS1 from urinary extracellular vesicles and decreased differential renal function in children with ureteropelvic junction obstruction

**DOI:** 10.1186/s12882-024-03592-0

**Published:** 2024-05-08

**Authors:** Lingyun Bu, Lingling Zhang, Xiaoqing Wang, Guoqiang Du, Rongde Wu, Wei Liu

**Affiliations:** 1 Department of Pediatric Surgery, Shandong Provincial Hospital Affiliated to Shandong First Medical University, 324Jingwu Road, Jinan, China; 2 Department of Minimally Invasive Urology, Jinan Children’s Hospital, Jinan, China

**Keywords:** Ureteropelvic junction obstruction, Urinary extracellular vesicles, NDUFS1, Differential renal function

## Abstract

**Background:**

Ureteropelvic junction obstruction (UPJO) is the most common cause of pediatric congenital hydronephrosis, and continuous kidney function monitoring plays a role in guiding the treatment of UPJO. In this study, we aimed to explore the differentially expressed proteins (DEPs) in the urinary extracellular vesicles(uEVs) of children with UPJO and determine potential biomarkers of uEVs proteins that reflect kidney function changes.

**Methods:**

Preoperative urine samples from 6 unilateral UPJO patients were collected and divided into two groups: differential renal function (DRF) ≥ 40% and DRF < 40%.We subsequently used data-independent acquisition (DIA) to identify and quantify uEVs proteins in urine, screened for DEPs between the two groups, and analyzed biofunctional enrichment information. The proteomic data were evaluated by Western blotting and enzyme-linked immunosorbent assay (ELISA) in a new UPJO testing cohort.

**Results:**

After one-way ANOVA, a *P* adj value < 0.05 (*P*-value corrected by Benjamin–Hochberg) was taken, and the absolute value of the difference multiple was more than 1.5 as the screening basis for obtaining 334 DEPs. After analyzing the enrichment of the DEPs according to Gene Ontology (GO) and Kyoto Encyclopedia of Genes and Genomes (KEGG) enrichment combined with the protein–protein interaction (PPI) network results, we selected nicotinamide adenine dinucleotide-ubiquinone oxidoreductase core subunit S1 (NDUFS1) for further detection. The expression of NDUFS1 in uEVs was significantly lower in patients with DRF < 40% (1.182 ± 0.437 vs. 1.818 ± 0.489, *P* < 0.05), and the expression level of NDUFS1 was correlated with the DRF in the affected kidney (*r* = 0.78, *P* < 0.05). However, the NDUFS1 concentration in intravesical urine was not necessarily related to the change in DRF (*r* = 0.28, *P* = 0.24).

**Conclusions:**

Reduced expression of NDUFS1 in uEVs might indicate the decline of DRF in children with UPJO.

**Supplementary Information:**

The online version contains supplementary material available at 10.1186/s12882-024-03592-0.

## Background

Hydronephrosis is a common malformation in pediatric urological diseases. Ureteropelvic junction obstruction (UPJO) is the most common cause of pediatric congenital hydronephrosis and can lead to irreversible kidney function damage in severe cases [1]. The overall incidence of UPJO is 1:1500, with a male-to-female ratio of 2:1, and 60% to 80% of UPJO cases are usually unilateral [2]. Indications for surgical intervention include clinical symptoms, worsening hydronephrosis on serial kidney ultrasound, and progressive deterioration of differential renal function (DRF) of less than 40% on serial diuretic nephrography [3]. Therefore, monitoring DRF in children with UPJO plays a crucial role in predicting the progression of the disease throughout its course [4].

With the increasing use of biomarkers in the diagnosis and treatment of diseases in recent years, accurate and noninvasive molecular markers are receiving increasing attention[5]. Extracellular vesicles (EVs) are less than 200 nm diameter vesicles released into the extracellular environment by functional cells through exocytosis; these vesicles have a phospholipid bilayer structure [6]. EVs are rich in proteins, nucleic acids, lipids, etc., and are closely related to cell growth and differentiation, angiogenesis, immune regulation, and inflammatory processes [7]. EVs not only are involved in cellular communication and transmission of genetic information [8] but also reflect and regulate normal physiological and abnormal pathological processes [9]. The urine contains large amounts of EVs, and almost all intrinsic kidney cells, such as kidney tubular epithelial cells, podocytes, and other cells of the urinary tract, are capable of secreting EVs [10]. In addition, the presence of urinary EVs (uEVs) has been studied as an indicator of kidney function damage [11]. However, there are no relevant studies on the ability of uEVs to predict postoperative kidney function recovery in children. In this study, we used data-independent acquisition (DIA) to identify and quantify EVs proteins in the urine of children with UPJO with different DRF strains, screened for differentially expressed proteins (DEPs) and performed bioinformatics to analyze the functions of these proteins. We identified the DEPs in the uEVs of children with UPJO and explored whether these uEVs proteins could be used as potential biomarkers reflecting DRF changes in these patients.

## Methods

### Participants and urine collection

Patients with SFU grade IV unilateral UPJO who underwent pyeloplasty in our single center between 2019 and 2022 were retrospectively reviewed. The cases with a solitary kidney, vesicoureteral reflux, kidney dysplasia, duplex kidney, or other urinary malformations and those who had concomitant urinary tract infection and incomplete clinical data were excluded. Preoperatively, all children were evaluated with diuretic renography using 99mTc-DTPA to determine the DRF. Urine was obtained from the patient’s bladder preoperatively, placed in culture flasks, and stored at -80 °C.The indications for pyeloplasty were worsening hydronephrosis and DRF of the afected kidney < 40%.The presentation with symptoms (pain, infection) was also considered indications for surgery.

There are total 26 patients were included in the study. According to the DRF cut-off value of 40%, they were divided into DRF ≥ 40% group and DRF < 40% group, with 13 patients in each group. Three samples were selected from each group for DIA and 20 samples were used for validation.

### Exosome isolation and purification

The uEVs were isolated and purified by ultracentrifugation [12]. Next, 25 ml of urine was centrifuged at 4 °C and 2000 × g for 15 min to remove cellular debris and bacteria, and the supernatant was collected. The supernatant was centrifuged at 10,000 × g for 30 min at 4 °C. The supernatant was collected in ultrafiltration centrifuge tubes (Millipore, USA) and centrifuged (Hitachi, Japan) at 4 °C and 100,000 × g for 4 h to obtain an uEVs pellet. The pellet was resuspended in phosphate-buffered saline and stored at -80 °C. The morphology of exosome particles was observed by JEM-100CXII transmission electron microscopy (Jeol, Japan), and the particle size distribution was analyzed by nanoparticle tracking analysis (NTA) using specific setup parameters. The parameters for NTA capture setting were as follows: Camera Type (sCMOS), Laser Type (Blue488), Camera Level (11), Slider Shutter (890), Slider Gain (125), FPS (25.0), Number of Frames (1498), Temperature (19.3—19.3℃), Viscosity (Water) 1.017—1.018 cP, Dilution factor (5.1 × 10e1). Data was analyzed by NTA 3.4 Build 3.4.4 (NanoSight Model NS300, Malvern Instruments, NanoSight Ltd., USA), Detect Threshold (5), Blur Size (Auto), Max Jump Distance (Auto: 10.9—11.3 pix). EVs preparation was done in compliance with the MISEV 2023 guidelines [13] (see Additional file 1).

### Proteomic analysis and data processing

Two group samples were selected, and each group was represented by three biological replicates. The preparation included protein denaturation, reduction, and alkylation as well as tryptic digestion and peptide cleanup, nano-HPLC‒MS/MS analysis, and data analysis. All six samples were processed by DIA individually to assess the proteome differences. MS1 and MS2 data were all acquired, and samples were acquired in random order. The iRT kit (Ki3002, Biognosys AG, Switzerland) was added to all of the samples to calibrate the retention time of extracted peptide peaks. The mass spectrometer was run under DIA mode with a hybrid data strategy. The statistical analysis of the DIA dataset was performed by Spectronaut 15(Biognosys AG, Switzerland) including data normalization and relative protein quantification. Hierarchical clustering analysis was performed with the pheatmap package (https://CRAN.R-project.org/package=pheatmap). Volcano plots were created using the ggplot2 package (http://ggplot2.org). Blast2GO version 5 was used for functional annotation, and GOATOOLS was used to perform GO enrichment analysis. Pathway analysis was performed with KOBAS (http://kobas.cbi.pku.edu.cn/). A heatmap was generated with https://www.bioinformatics.com.cn, an online platform for data analysis and visualization. A PPI network was constructed by using STRING v10 (www.string-db.org).

### Western blot analysis

The cell precipitates and uEVs pellets were dissolved in RIPA buffer (Bioss, China), phenylmethanesulfonyl fluoride (PMSF) (Solarbio, China), and phosphatase inhibitor cocktail II (MedChemExpress, USA) were added, and the protein supernatant was collected after centrifugation. The extraction protein concentration was determined with a bicinchoninic acid protein assay kit (Servicebio, China). Equal amounts of proteins were separated via 10% sodium dodecyl sulfate–polyacrylamide gel electrophoresis (Vazyme, China). The proteins were subsequently transferred to 0.2 µm polyvinylidene difluoride membranes (Millipore, USA), which were blocked with 5% bovine serum albumin for 1 h and then incubated with primary antibodies overnight at 4 °C. The primary antibodies used were rabbit anti-CD63 (Abcam/ab92726, USA), rabbit anti-TSG101 (Abcam/ab2788, USA), rabbit anti-calnexin (Abcam/ab22595, USA), rabbit anti-NDUFS1 (ABclonal/A21192, China), and rabbit anti-GAPDH (Proteintech/10494–1-AP, China). Afterward, the membranes were incubated with a secondary antibody conjugated to horseradish peroxidase (Proteintech, China) for 1 h at room temperature and then visualized with a chemiluminescence imaging system (General Electric, USA).

### Enzyme-linked immunosorbent assay analysis

Five hundred microliters of urine were collected from each patient and added to a sterile test tube. The collected urine was subsequently centrifuged at 4 °C for 10 min at 2500 rpm. The protein content in the samples was detected by the quinoline formic acid protein Assay kit (Servicebio, China), and the protein content of the samples was normalized. The supernatant was placed in a new EP tube and kept in a -80 °C freezer. An NDUFS1 enzyme-linked immunosorbent assay kit (Jianglai Biologicals, China) was used to measure the levels of NDUFS1 in whole urine.

### Data analysis

The experimental data were analyzed using the SPSS 27.0 Chinese package and plotted using GraphPad Prism 8. The normality test was performed using the Shapiro‒Wilk test, and the t-test or nonparametric rank sum test was used to compare the two groups. A *P* value < 0.05 indicated a statistically significant difference.

## Results

### Isolation and identification of urinary extracellular vesicles

**Fig. 1:**
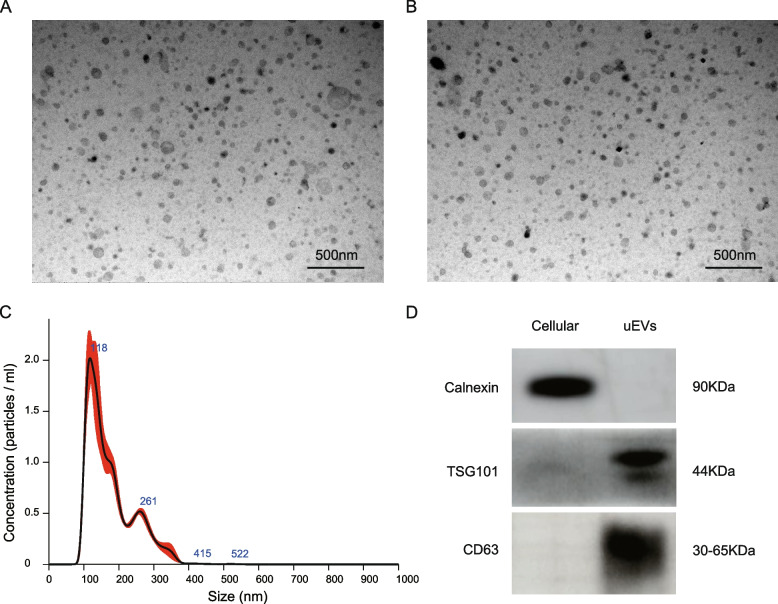
Identification of urinary extracellular vesicles. **A**, **B** Transmission electron microscopy showed that the uEVs had a discoid vesicle-like structure with an intact lipid bilayer. **C** Nanoparticle tracking analysis: the size range of the particles. **D** Western blot experiments showed that calnexin was not expressed in uEVs but that TSG101 and CD63 were expressed in uEVs. Full-length blots/gels are presented in Additional file 2

EVs were separated from urine by ultracentrifugation. Transmission electron microscopy revealed that the uEVs had a discoid vesicle-like structure with an intact lipid bilayer. NTA revealed that the vast majority of the vesicles were 40–200 nm in diameter. The uEVs marker proteins TSG101 and CD63 were expressed in the vesicles, but calnexin was not expressed in the vesicles. The successful isolation and purification of uEVs were confirmed (Fig. 1).

### Proteomic analysis of urinary extracellular vesicles in children with UPJO

**Fig. 2:**
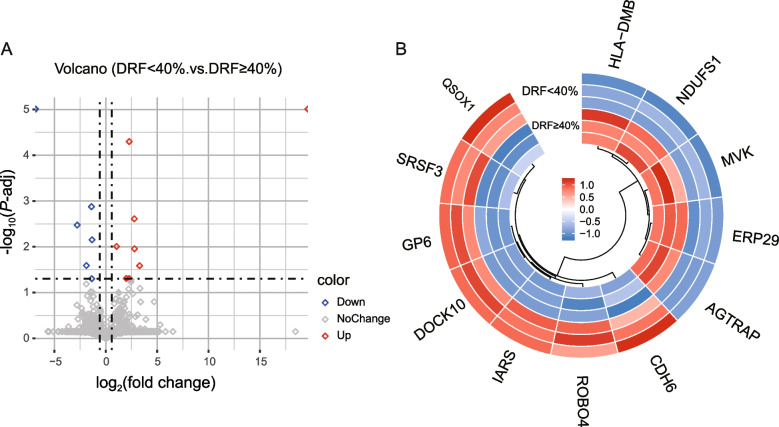
Proteomic analysis of urinary extracellular vesicles. **A** Volcano plot of proteins with FDR values < 0.05 and absolute fold change values > 1.5. **B** Clustering diagram of the major differentially expressed proteins between samples

A total of 3153 proteins were identified in this study; 3037 proteins were identified in DRF ≥ 40%, and 2947 proteins were identified in DRF < 40%. However, the first 3 peptides filtered by a 1% Q cutoff were used to calculate the number of major groups in this study. After one-way ANOVA was performed and the Benjamin–Hochberg method was used to correct the *P* value, a total of 334 differentially expressed proteins were ultimately identified with a false discovery rate (FDR) < 0.05 and an absolute fold change > 1.5 (Fig. 2A). Among them, 123 proteins were upregulated and 211 proteins were downregulated in patients with DRF < 40% (see Additional file 3). We clustered the proteins that appeared on the volcano plot (Fig. 2B), and the details are shown in Table 1.

**Table 1:** Major differential proteins in uEVs in children with unilateral SFU grade IV UPJO (DRF < 40% vs. DRF ≥ 40%)

Protein Accessions	Protein Abbreviation	Protein Descriptions	log2FoldChange	*P* Value	*P *adj value
P28331	NDUFS1	nicotinamide adenine dinucleotide -ubiquinone oxidoreductase core subunit S1	-2.7845	0.000346	0.003348
Q6RW13	AGTRAP	Type-1 angiotensin II receptor-associated protein	-1.8953	0.002688	0.025679
P30040	ERP29	Endoplasmic reticulum resident protein 29	-1.39506	0.000137	0.00133
Q03426	MVK	Mevalonate kinase	-1.37295	0.005269	0.049744
P28068	HLA-DMB	HLA class II histocompatibility antigen, DM beta chain	-1.33914	0.000728	0.007022
Q8WZ75	ROBO4	Roundabout homolog 4	1.049631	0.001024	0.009844
O00391	QSOX1	Sulfhydryl oxidase 1	1.996389	0.005161	0.049011
P55285	IARS	Cadherin-6	2.277985	0.005223	0.04945
P41252	CDH6	Isoleucine–tRNA ligase, cytoplasmic	2.277985	5.17E-06	5.04E-05
Q9HCN6	GP6	Platelet glycoprotein VI	2.776104	0.000252	0.002446
P84103	SRSF3	Serine/arginine-rich splicing factor 3	2.790772	0.001152	0.01104
Q96BY6	DOCK10	Dedicator of cytokinesis protein 10	3.286881	0.002715	0.025863

### Bioinformatics analysis of differentially expressed proteins

**Fig. 3:**
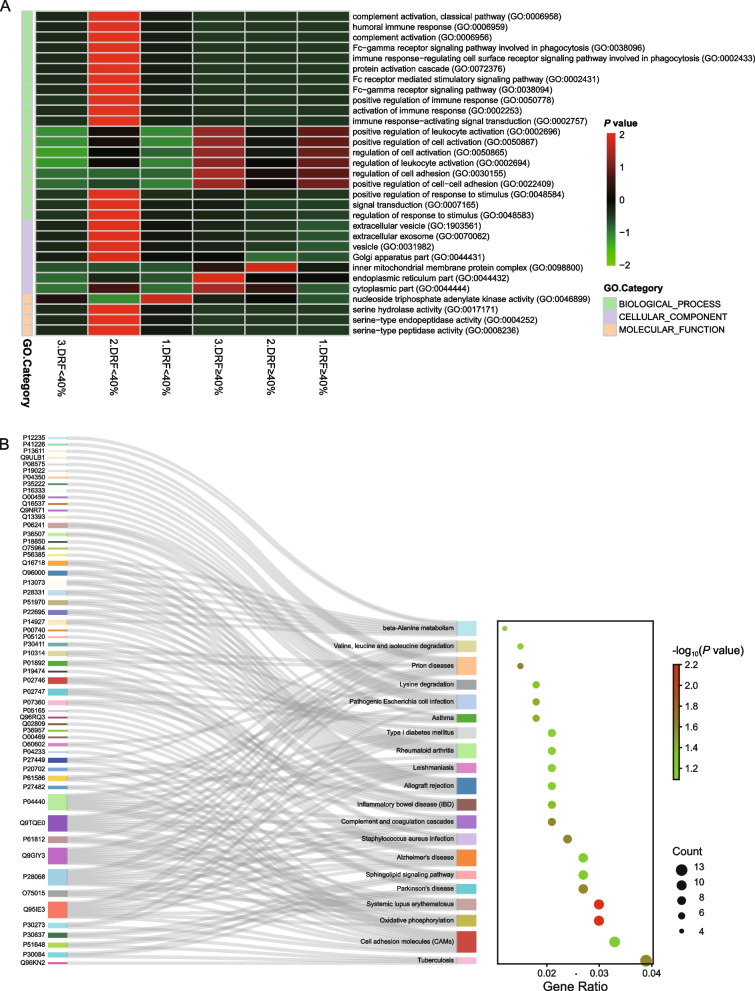
Bioinformatics analysis of the differentially expressed proteins. **A** Cluster map of the GO enrichment analysis results for the DEPs. The color from green to red indicates higher expression. The color bars on the left are used to distinguish biological processes, cellular components, and molecular functions. **B** DEPs-based KEGG concentration bubble map. The left side of this figure is the Sankey plot, representing the proteins contained in each pathway, and the right side is the conventional bubble plot, where the bubble size indicates the number of proteins to which the pathway belongs, and the bubble color indicates the *P*-value

**Fig. 4:**
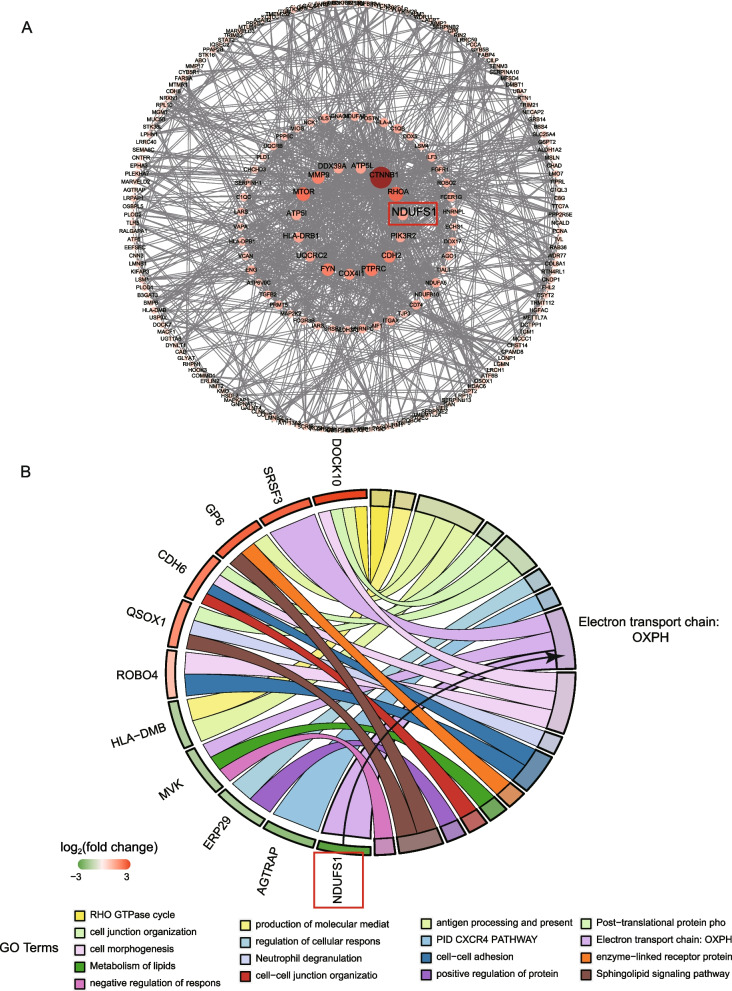
Screening of hub protein. **A** PPI network diagram of the differentially expressed proteins according to the degree algorithm. Darker colors and larger bubbles represent greater connectivity. **B** GO enrichment STRING diagram of major differentially expressed proteins. The enrichment results are presented using chordal plots, with genes on the left, GO terms on the right, and the middle line indicating affiliation. The proteins ranked by − log_10_(*P*) value are shown by the Circro diagrams

GO functional enrichment showed that the upregulated proteins in the DRF < 40% group were involved mainly in biological processes such as activation, regulation, and signal transduction of the immune response, while the downregulated proteins were involved mainly in biological processes such as activation of leukocytes, regulation of cell adhesion and signal transduction. These DEPs  mainly exercised enzymatic activity, such as serine hydrolase, kinase, and endopeptidase activity, and thus participated in the activation and transduction of related signals (Fig. 3A). Pathway enrichment analysis of the screened DEPs using the KEGG database revealed that the DEPs were concentrated in the signaling pathways associated with tuberculosis, systemic lupus erythematosus, oxidative phosphorylation, cell adhesion, and Parkinson's disease (Fig. 3B). The screened DEPs were used to construct PPI network graphs with the STRING database and mapped in Cytoscape using the degree algorithm. These proteins were biologically well linked, and the differentially expressed proteins at key nodes and with the highest connectivity were visualized (Fig. 4A). After analyzing the GO and KEGG enrichment results for the differentially expressed proteins, we selected NDUFS1, which had a large fold change and a small FDR value and was the key node with high connectivity, as the object of further study (Fig. 4B).

### Expression of NDUFS1 in urinary extracellular vesicles of children with UPJO is associated with DRF

**Fig. 5:**
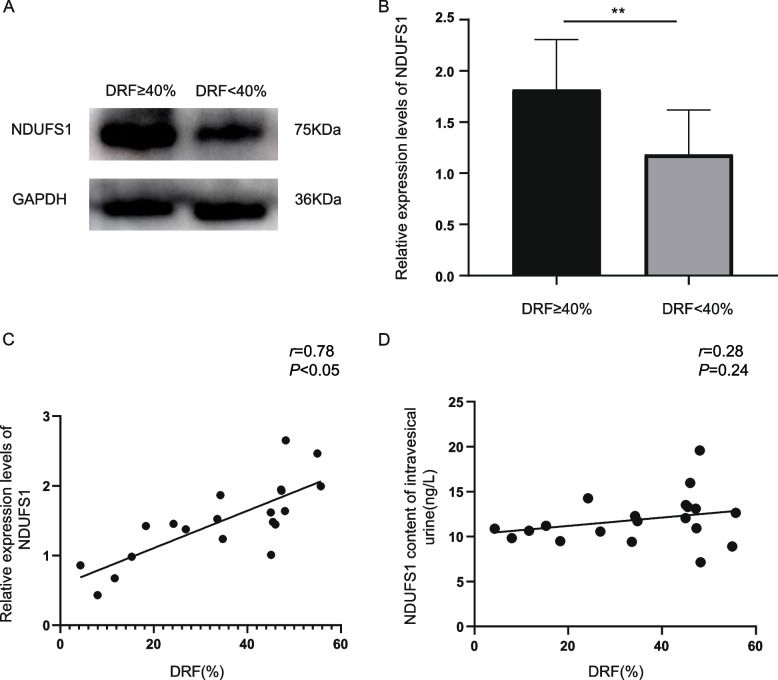
Relationship between NDUFS1 expression and DRF in children with UPJO. **A**, **B** Decreased expression of uEVs-derived NDUFS1 in children with unilateral UPJO and an SFU level of 4 with DRF < 40%. Full-length blots/gels are presented in Additional file 2. **C** Correlation between the relative uEVs expression levels of NDUFS1 and DRF. **D** Correlation between urinary NDUFS1 levels in the bladder and changes in DRF. *P < 0.05, **P < 0.01

We validated the results of the DIA analysis by measuring the protein levels of NDUFS1 in the uEVs of 20 children with UPJO. The general characteristics and specific groupings of the children are shown in Table 2. The results showed that the protein level in the DRF ≥ 40% group (1.818 ± 0.489) was significantly greater than that in the DRF < 40% group (1.182 ± 0.437) (*P* < 0.05; Fig. 5A, B), which was consistent with the results of mass spectrometry analysis. We also determined the relationship between the relative expression levels of NDUFS1 in bladder uEVs and DRF (*r* = 0.78, *P* < 0.05) (Fig. 5C), revealing a correlation between uEVs-derived NDUFS1 levels and DRF. We further evaluated the level of NDUFS1 in intravesical urine using an enzyme-linked immunosorbent assay kit and showed that the level of NDUFS1 in intravesical urine was not necessarily associated with changes in DRF (*r* = 0.28, *P* = 0.24) (Fig. 5D).

**Table 2:** Characteristics of the UPJO patients

Variable	DRF ≥ 40% (*n* = 10)	DRF < 40% (*n* = 10)	*P*-value
Gender (boy/girl)	9/1	7/3	0.58
Age at examination (months)	54.10 ± 59.03	39.5 ± 49.95	0.64
Laterality (right/left)	2/8	3/7	> 0.99
Blood urea nitrogen(mmol/L)	4.30 ± 0.78	3.72 ± 1.15	0.24
Serum creatinine(μmol/L)	32.01 ± 8.81	33.75 ± 18.35	0.88
Anteroposterior diameter(mm)	35.40 ± 27.35	42.56 ± 16.62	0.51
Parenchymal thickness(mm)	6.41 ± 2.70	4.33 ± 2.45	0.10
DRF (%)	48.31 ± 3.89	21.11 ± 11.27	< 0.05

Values are means ± standard deviations

## Discussion

Due to the obstruction of the kidney outflow tract in children with UPJO, the pressure in the kidney pelvis gradually increases, which can lead to severe impairment of kidney function, which sometimes requires surgery [14]. Therefore, monitoring kidney function, especially DRF, in children with UPJO plays a crucial role in predicting the progression of the disease [4]. At present, radionuclide renal dynamic imaging is the most commonly used method to evaluate renal function and other monitoring methods are rarely studied. Urine is a treasure trove of biomarkers that have been explored by researchers. Recently, Chen et al. used tandem mass spectrometry to perform proteomic analysis of bladder and renal pelvis urine from children with unilateral UPJO (DRF < 40%), combined with urine from healthy controls, and found increased concentrations of fetuin-A and α1-acid glycoprotein 1 in the urine of the disease group [15]. More notably, a study has found a strong correlation between bladder uEVs-derived matrix remodeling associated protein 5 (MXRA5) and DRF in children with UPJO, suggesting that uEVs may be more significant than biomarkers provided by whole urine [11].

EVs target receptor cells via surface molecules, fuse with the cell membrane of target cells via endocytosis and/or phagocytosis, and deliver specific loaded proteins, nucleic acids, lipids, and other information to the target cells, thereby altering the physiological state of the cells [16]. In this study, uEVs proteins from children with unilateral UPJO at the SFU grade IV were analyzed using DIA technology to identify proteins related to changes in DRF that are involved in UPJO disease progression. Analysis of the GO and KEGG enrichment results of the differentially expressed proteins combined with the PPI network revealed that the key node NDUFS1, which had a large fold change, a small FDR value, and high connectivity, was the object of further study. In our study, NDUFS1 expression was downregulated in the uEVs of children with DRF < 40%, as verified by Western blotting. The changes in NDUFS1 are positively correlated with the changes in DRF, which can reflect the changes in DRF in UPJO to a certain extent.

NDUFS1, a key iron/sulfur cluster in complex I, is the largest subunit in mitochondrial oxidative respiratory chain complex I. NDUFS1 is involved mainly in oxidative phosphorylation and electron respiratory chain transfer. It has NADH dehydrogenase and oxidoreductase activity and catalyzes the oxidation of NADH and the reduction of ubiquinone, using the energy generated by this process to transfer protons to the inner mitochondrial membrane. Mitochondrial dysfunction is an important cause of many diseases, such as neuromuscular diseases and cardiovascular diseases, and when oxidative stress occurs in mitochondria in vivo, NDUFS1 is inactivated, which results in the generation of large amounts of reactive oxygen species and other subsequent changes [17]. Mitochondrial damage plays a key role in acute kidney injury (AKI). Several studies have found that the expression of NDUFS1 is down-regulated in the inflammatory factor-induced AKI kidney and the transcriptome of mouse proximal renal tubular cells [18]. One study revealed that high levels of oxidative stress in the skin fibroblasts of patients with NDUFS1 mutations were accompanied by decreased complex I activity, decreased oxygen consumption, and increased glycolysis [19], and reduced expression of NDUFS1 in the myocardium may be an important cause of myocardial dysfunction and myocardial fibrosis [20]. Elkholi et al. showed that sequestration of NDUFS1 increases ROS production, which may lead to DNA damage and subsequent activation of the mitochondrial apoptotic pathway [21]. These findings may suggest that as kidney function declines, oxidative stress increases in the kidney, reflecting, in part, increased kidney fibrosis. However, in this study, the sample size was small, and only performed preoperative DRF analysis and uEVs protein quantitative analysis, whether NDUFS1 level can fully reflect the changes in renal function and guide the surgery needs further study.

## Conclusions

In this study, we detected changes in NDUFS1 content in bladder uEVs reflecting changes in DRF in both groups of unilateral UPJO patients, suggesting that uEVs derived NDUFS1 might be used as a potential biomarker for decreased DRF in children with UPJO.

### Supplementary Information

Supplementary Material 1.

Supplementary Material 2.

Supplementary Material 3.

## Data Availability

Data is provided within the manuscript or supplementary information files.

## Abbreviations

UPJO: Ureteropelvic junction obstruction
DRF: Differential renal function
NDUFS1: Nicotinamide adenine dinucleotide-ubiquinone oxidoreductase core subunit S1
DIA: Data-independent acquisition
GO: Gene Ontology
KEGG: Kyoto Encyclopedia of Genes and Genomes
PPI: Protein–protein interaction
ELISA: Enzyme-linked immunosorbent assay
DEP: Differentially expressed protein
uEVs: Urinary extracellular vesicles
