# Supplementary material for: Association between NDUFS1 from urinary extracellular vesicles and decreased differential renal function in children with ureteropelvic junction obstruction
Source: BMC Nephrol. 2024 May 8;25:158. doi: 10.1186/s12882-024-03592-0 (PMC11080270; doi:10.1186/s12882-024-03592-0)
Supplement: Supplementary file 1 — Supplementary Material 1. [file 12882_2024_3592_MOESM1_ESM.pdf]

MISEV 2023 guidelines compliance for uEVs

Section title	Evidence level	Recommendation	Our approach
Existing biobanks	High	Report as many parameters as possible.	We collected all below-mentioned parameters and determine if sample collection is appropriate for our research purpose (Table 2). Perform tests to determine urine quality, number and characteristics of EVs.
Storage of urine prior to processing	High	Longer storage time may lead to microbial growth, cell debris, sedimentation, and degradation of more labile biomolecules (e.g. RNA).	Morning urine samples were collected from the study subjects in sterile enzyme-free centrifuge tube and immediately placed in a 4 ° C refrigerator for use. Urine samples were processed within 12 hours.
	High	Freshly collected urine samples should be cooled promptly to avoid microbial growth or biomolecule degradation. Avoid freezing at this step.	
	Low	Some urinary analytes may be light sensitive (e.g. bilirubin, porphyrins); impact on uEVs is unknown. Use amber-colored or dark collection tubes.	Not applicable/not available
	High	The presence of cells, microbes as well as high levels of protein and other factors affect the purity and composition of uEV population. Use dipsticks to examine urine quality and identify sample outliers. Report	

		dipstick brand, tested parameters, and sample inclusion criteria and cutoffs.	
Preprocessing	Medium	Preservative might be affected by time and storage in collection container. If protease inhibitors are used at the time of collection, it is recommended that sample containers are prepared by adding protease inhibitor cocktail. Protease inhibitor cocktail aliquots should be kept frozen at -20°C for a maximum of 6 months.	Urine samples were processed within 12 hours by adding protease inhibitors (Beijing Solaibao Biotechnology Co., LTD.) to the collected urine at a 100:1 ratio.
	High	Freshly collected urine samples should be processed promptly to avoid microbial growth or biomolecule degradation. Consider addition of protease inhibitors or preservatives when fast processing (faster than 6 hours) is not possible (see above).	
	Medium	Centrifuge at a maximum of 800 x g to sediment cells and debris present in urine without damaging them. Report centrifuge and rotor model, G-force, volume, temperature, and duration.	Centrifuge: Thermo Scientific HeraeusCryofuge 8/16 Rotor Model: A27-8 x 50 fixed Angle turn head<br>G-force: 800g<br>Volume: 50mL<br>Temperature: 4℃<br>Duration: 15min
	Medium	Operator-dependent. Report volume. Report method (e.g. pipetting,	

		decanting).	
	Medium - High	Collection and storage of pellet and whole urine aliquots is recommended to monitor the uEVs purification process.	Not applicable/not available
Storage of urinary supernatant and uEVs	Medium	As samples may be used for several experiments, when possible, collect aliquots of different volumes to avoid repeated freeze/thawing. Large, up to 30 ml; Medium, 5 - 10 ml; Small, 1 - 2 ml.	Due to the limited urine samples, the samples were timely subjected to differential centrifugation to obtain EVs, dissolved with RIPA lysate, PBS, packaged, and stored at -80°Cfor subsequent experiments.
	Medium	Use max ¾ of container volume to accommodate sample expansion. Storage container should resist pH range of urine and not shed any particles. Low EV binding properties are generally beneficial.	
	Medium	Quick freezing is generally recommended to preserve biological specimens, but impact of freezing speed or cryoprotective agents on uEVs is unknown. Freeze quickly in -70°C freezer or snap freeze in liquid nitrogen. Report freezing method and sample volume.	
	Medium	Particle counts may decrease and lead to loss of	

		antigenicity of EV proteins after storage at -20°C, EV yield from samples stored at -20°Cmay be lower. Freeze immediately and store at -70°Cor lower.	
Defrosting	Low	Heating pad, water bath, incubator, room temperature, refrigerator. Standardize defrosting method and use the same technique for all samples.	For Western blotting assays, samples were removed from the -80°C freezer and thawed on ice. Avoid repeated freezing and thawing
	Low	The effect on thawing temperature on uEVs has not been studied extensively. However, high temperatures might affect heat labile biomolecules or lead to sediment formation.	
	Low	Longer thawing times may require addition of preservatives.	
Transportation of uEVs	Medium-High	EV quality and quantity diminish during long-term exposure at room temperature and during multiple freeze-thaw cycles. Use cooling system whenever possible. Preservatives can prevent protein/RNA breakdown and bacterial outgrowth. Transport uEVs and processed supernatant frozen (≤ -70°C) and whole urine at +4°C.	EVs obtained by differential centrifugation were collected into 1.5mL EP tubes (Corning, USA) and transported on dry ice for NTA, TEM, and DIA quantitative protein detection.

	Medium-High	uEV quality and quantity diminish with long-term storage at room temperature. Container leakage could introduce contamination. Inspect containers for integrity and damage.	
