# Supplementary figures and images for: Association between NDUFS1 from urinary extracellular vesicles and decreased differential renal function in children with ureteropelvic junction obstruction
Source: BMC Nephrol. 2024 May 8;25:158. doi: 10.1186/s12882-024-03592-0 (PMC11080270; doi:10.1186/s12882-024-03592-0)

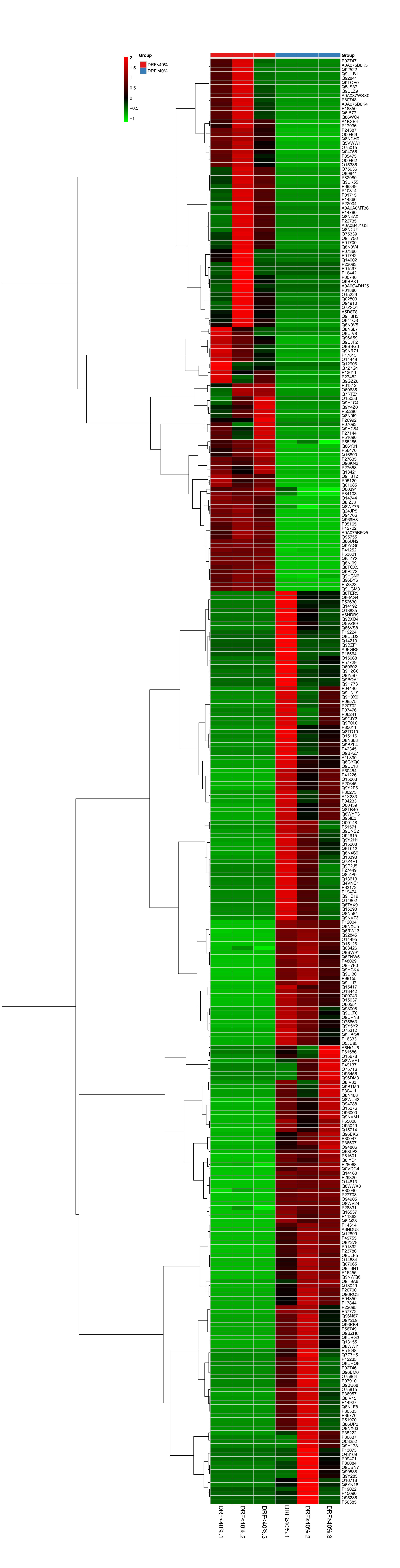

Supplement: Supplementary file 3 — Supplementary Material 3. [file 12882_2024_3592_MOESM3_ESM.pdf]
